# Hedgehog Pathway Signaling Regulates Human Colon Carcinoma HT-29 Epithelial Cell Line Apoptosis and Cytokine Secretion

**DOI:** 10.1371/journal.pone.0045332

**Published:** 2012-09-19

**Authors:** Agnes N. Yoshimoto, Claudio Bernardazzi, Antonio José V. Carneiro, Celeste C. S. Elia, Cesonia A. Martinusso, Grasiella M. Ventura, Morgana T. L. Castelo-Branco, Heitor S. P. de Souza

**Affiliations:** 1 Laboratório Multidisciplinar de Pesquisa, Universidade Federal do Rio de Janeiro, Rio de Janeiro, Brazil; 2 Serviço de Gastroenterologia, Departamento de Clínica Médica, Universidade Federal do Rio de Janeiro, Rio de Janeiro, Brazil; 3 Unidade de Microscopia Confocal, Universidade Federal do Rio de Janeiro, Rio de Janeiro, Brazil; 4 Laboratório de Imunologia Celular, Instituto de Ciências Biomédicas, Universidade Federal do Rio de Janeiro, Rio de Janeiro, Brazil; National Cancer Center, Japan

## Abstract

The Hedgehog (Hh) pathway is involved in embryogenesis and physiologic processes including cell survival and proliferation. We used the HT-29 and other human colon carcinoma cell lines to investigate Hh signaling and biological functions in colonic epithelial cells. HT-29 cells were cultured under different conditions and exposed to various stimuli. The expression of Hh pathway components and related genes and proteins were assessed by real-time PCR and immunofluorescence. Viability, apoptosis and cell proliferation were measured by the MTT assay, Annexin-V/7-AAD staining and BrdU uptake, respectively. Chemokines production was measured by ELISA in culture supernatants. *Indian* and *Sonic Hh* mRNA levels and the downstream transcription factors *Gli-1* and *Gli-2* increased following treatment with Hh agonists and butyrate, but decreased upon exposure to cyclopamine or GANT61. *BMP4* and *BMP7* expression increased after stimulation with Hh agonists. Gli-1 protein expression increased after Hh agonists and decreased following cyclopamine. Exposure to Hh agonists promoted β-catenin reduction and subcellular redistribution. Levels of IL-8 and MCP-1 decreased upon exposure to Hh agonists compared to Hh antagonists, LPS, IFN-γ or EGF. Monocyte chemotaxis decreased upon exposure to supernatants of HT-29 cells treated with Shh compared to Hh antagonists, LPS and IFN-γ. Cellular incorporation of BrdU and cell viability decreased following Hh blockade. Hh agonists abrogated the anti-CD95 induced apoptosis. Hh pathway is a key controller of colon cancer cells, as demonstrated by its effect in dampening inflammatory signals and antagonizing apoptosis. The differential expression of Hh components may underlie abnormalities in the local immune response and in epithelial barrier integrity, with potential homeostatic implications for the development of colonic inflammation and malignancies.

## Introduction

An essential physiologic function of the intestine is the maintenance of a protective barrier against potentially harmful gut luminal contents. The first line of defense in this barrier is constituted by a single layer of epithelial cells that continuously renew and interact with the lumen and the lamina propria immune cells [1], [2]. Control of cell renewal within the epithelial layer depends on a dynamic equilibrium between cell proliferation and apoptosis, whereas imbalance between the two mechanisms is known to result in cancer [3] and also in inflammatory conditions [4].

The Hedgehog (Hh) pathway of morphogenic proteins is known to participate in several physiologic cellular processes. Hh signaling has been associated with embryogenesis [5], patterning of a variety of organs [6], adult tissue homeostasis and repair [7], regulation of the epithelial-to-mesenchymal transition [8], and the control of cell survival and proliferation [9]. Activation of the canonical Hh signaling pathway involves the binding of Hh ligands to the membrane receptor Patched (PTCH1), resulting in the activation of the signaling cytoplasmic molecule Smoothened (SMO). Once released from PTCH-mediated suppression, SMO activates the Gli zinc finger transcription factors that ultimately regulate downstream Hh target genes [6]–[8], [10].

The canonical Hh signaling cascade has also been shown to play a role in the normal gastrointestinal development, where it regulates the differentiation of normal intestinal villi [11], and the adjacent mesenchymal stromal cells [12]. In the normal adult gastrointestinal tract, induction of the Hh pathway appears to protect the differentiated epithelial cells of the villous surface, counteracting the canonical Wnt signaling in the basal cells of the crypt [13]. Although aberrant activation of the Hh pathway has been demonstrated in the oncogenesis of human esophageal, gastric, and pancreatic cancers [14], [15], its role in colon cancer has not been sufficiently clarified.

The majority of sporadic and hereditary human colorectal tumors are believed to originate from constitutive activation of Wnt signaling by mutation of the APC or β-catenin genes [16], whereas the Hh pathway would oppose the proliferative effect of Wnt pathway on differentiated colonocytes [13]. Recently, there has been evidence of the potential involvement of Hh signaling in cancer invasiveness [17], in colonic carcinogenesis [18], [19], and in the metastatic dissemination of colorectal cancer [20]. Nevertheless, approaches to elucidate the role of the Hh pathway in cancers are still limited, in particular in colon cancer where data have been controversial [21], [22].

In order to investigate Hh signaling and its potential biological functions in colonic epithelial cells, we used the epithelial HT-29 and other human colon carcinoma cell lines under various experimental conditions. We demonstrated the presence and modulation of several Hh components and that Hh pathway activation has anti-inflammatory and anti-apoptotic effects on colon cancer cells.

## Methods

### Cell Cultures

Human colon adenocarcinoma cell line (HT-29) was obtained from the American Type Culture Collection (ATCC, Rockville, MD, USA), and maintained according to the ATCC’s instructions. Cells were grown as monolayers in Dulbecco’s modified Eagle’s medium (DMEM) (Invitrogen Gibco, New York, NY, USA). Cells were plated in 25 cm^2^ culture flasks with DMEM supplemented with heat inactivated 10% fetal bovine serum (FBS) (Invitrogen Gibco, New York, NY, USA), penicillin (10,000 U/mL), streptomycin (100 µg/mL), 4.5 g/L glucose and L-glutamine, (all from Sigma-Aldrich, St. Louis, MO, USA) at 37°C in a humidified atmosphere with 5% CO_2_. After growth to confluence, cells were trypsinized and resuspended in DMEM supplemented with 2.5% FBS. Part of the cells were frozen and maintained at −80°C. Cells were then seeded at a density of 1**×**10^6^ cells/mL and treated specifically for each experiment in either 6-well, 24-well or 96-well microplates, as needed. Culture supernatants were collected and maintained at −80°C for further chemokine measurements. In parallel, cells were plated onto eight-chamber slides (Lab Tek Nunc, Naperville, IL, USA) for immunofluorescence staining.

### HCT8, HCT116, and Caco-2 Human Colon Cancer Cell Lines

Data regarding all experiments with these cells are presented in the Supporting Information S1 session.

### Reagents and Treatments

Recombinant human N-Shh was purchased from R&D Systems (Minneapolis, MN, USA), and used at 500 ng/mL; cyclopamine (Sigma-Aldrich, St. Louis, MO, USA) was used as a selective chemical hedgehog inhibitor, and diluted in dimethyl sulfoxide (DMSO) (control vehicle) (Merck, Darmstadt, Germany) at 2 µM; GANT61 (Tocris Bioscience, Bristol, UK), a small molecule that acts downstream of cyclopamine to inhibit GLI transcription, diluted in DMSO at 5 µM; chemical hedgehog agonist purmorphamine (Alexis Biochemicals, Plymounth, PA, USA) was diluted in DMSO at 2 µM; sodium butyrate was added to cultures at 2.5 µM, while interferon-gamma (IFN-γ) and lipopolysaccharide (LPS) were used at 2 ng/mL (all from Sigma-Aldrich, St. Louis, MO, USA); recombinant human epithelial growth factor (rhEGF) was obtained from Peprotech (Rocky Hill, NJ, USA), and used at 20 ng/mL.

### Indirect Immunofluorescence Staining and Confocal Laser Microscopy

Cells were seeded onto eight-chamber slides, fixed, permeabilized, and incubated for 2 h at room temperature with 2.5% bovine serum albumin (BSA), 2.0% skimmed milk, 8.0% fetal bovine serum (FBS) blocking buffer under shaking. Slides were rinsed once with PBS and 0.05% Tween 20 and then incubated with appropriately diluted primary antibodies in PBS. Cells were incubated with anti-Gli-1 rabbit antibody (Santa Cruz Biotechnology, Inc., Santa Cruz, CA, USA), and anti-β-catenin mouse antibody (Millipore, Bedford, MA, USA) for 1 h at room temperature. After incubation, the slides were rinsed three times and incubated with Alexa® 488 conjugated anti-rabbit IgG, and Alexa® 633 conjugated anti-mouse IgG (all from Molecular Probes, Eugene, OR, USA) for 30 min. at room temperature. Sections from each sample were incubated with either PBS alone or secondary antibody and served as negative isotype controls. Slides were air-dried, fixed for 5 min in a 1% paraformaldehyde solution, and mounted in an antifading medium containing 4',6-diamidino-2-phenylindole (DAPI) (Vector Labs, Inc., Burlingame, CA, USA). Expression and localization of the proteins were observed with a Leica TCS-SP5 AOBS confocal laser scanning microscope (Leica, Heidelberg, Germany), for capturing representative images of each sample.

### RNA Isolation and cDNA Synthesis

The expression levels of selected genes were validated by qRT-PCR. Thus, HT-29 cells were either untreated (vehicle control, 0.2% DMSO), or treated with cyclopamine, GANT61, purmorphamine, butyrate, LPS, EGF, or IFN-γ, for 24 hr at 37°C, dissolved in DMSO-containing medium. Total RNA isolation from cultured cells was performed using SV Total RNA isolation systems (Promega, Madison, WI, USA), following the manufacturer’s protocol. The Nanodrop 2000 UV-Vis Spectrophotometer (Thermo Scientific, Wilmington, DE, USA) was used for quantifying and determining the RNA purity of samples. Equal amounts of total RNA were reverse transcribed using RT2 First Strand Kit (SABiosciences, Frederick, MD, USA).

### Quantitative Real-Time PCR (qRT-PCR)

To quantify the changes in mRNA levels, real-time RT-PCR was performed on the ABI Prism 7500 (Applied Biosystems, Foster City, CA, USA) using RT2 Real Time ™ SYBR Green/Rox PCR Master Mix (SABiosciences, Frederick, MD, USA). For this purpose, we used a customized commercially available RT2 Profiler PCR Array for detecting *IHH, SHH, GLI-1, GLI-2, GLI-3, PTCH1, SMO, HHIP, WNT1, BMP4,* and *BMP7* genes (SABiosciences, Frederick, MD, USA). Levels of mRNA were normalized to the expression of glyceraldehydes phosphate dehydrogenase (*GAPDH*), *beta-actin*, and ribosomal protein L32 (*RPL32*), control genes. For data analysis the ΔΔCt method was used; determining the fold change for all target genes in each sample with 95% confidence. Q-RT-PCR for each gene was determined in duplicate, and each experiment was repeated at least three times. A positive value indicates gene up-regulation and a negative value indicates gene down-regulation. PCR cycles were performed according to the manufacturer's instructions.

### Chemokine Measurements

Culture supernatants were used for measuring the extra cellular concentration of the chemokines IL-8 (Bender MedSystems, Vienna, Austria) and MCP-1 (e-Bioscience, San Diego, CA, USA) by commercial sensitive enzyme-linked immunosorbent assays (ELISA) method. The total protein content of cell monolayers was estimated by the Pierce® BCA protein assay kit (Thermo Scientific, Rockford, IL, USA), and used for normalizing the results. The minimum detectable concentration of human IL-8 and MCP-1 was less than 5.0 ng/L.

### Isolation of Human Monocytes from Peripheral Blood

Monocytes were obtained from peripheral blood of three healthy human volunteers, and peripheral blood mononuclear cells (PBMCs) were layered over a Ficoll-Hypaque cushion, and centrifuged at 400 *g* for 20 min. PBMCs were harvested at the interface and washed twice with PBS and resuspended in RPMI 1640 medium supplemented with 10% heat-inactivated FBS (Gibco-Invitrogen, Carlsbad, CA, USA), 100 U/ml penicillin, and 100 µg/ml streptomycin (Sigma Chemical Co., St. Louis, MO, USA) at 37°C in a humidified atmosphere with 5% carbon dioxide. PBMCs were then plated onto a 25 cm^2^ culture flask, and monocytes were allowed to attach to the surface for 24 h. The adherent monocytes were washed twice with ice-cold Hank’s balanced solution and detached by trypsinization. The recovered cells were centrifuged at 2000 rpm and resuspended in plain RPMI 1640 medium supplemented with 2.5% FCS. Cell count and viability were determined by Trypan blue staining.

### Monocyte Chemotaxis Assay

Monocytic chemotactic activity was determined using the QCM Chemotaxis Cell Migration Assay (Millipore Corporation, Billerica, MA, USA) fitted with polycarbonate membrane of 5-µm pore size. Human monocytes (1×10^5^ cells) were incubated with 2.5% FBS complete medium in the upper chambers, while 24 h culture supernatants of HT-29 cells treated with either DMSO (control), rShh, cyclopamine, GANT61, IFN-γ, or LPS, were added to the lower chambers. Additional control wells received either 20 ng/ml of recombinant monocyte chemotactic protein-1 (rMCP-1), or anti-MCP-1 antibody (e-Bioscience, San Diego, CA, USA), at the bottom chambers of the plate. Cells were allowed to incubate at 37°C for 4 h, after which cell density in the bottom chamber was determined by a colorimetric assay, read in a 96-well plate at 560 nm.

### Assessment of Cell Proliferation and Viability

For the assessment of cell proliferation, we used the BrdU Cell Proliferation Assay Kit (Chemicon International, Temecula, CA, USA). To minimize spontaneous cell death, HT-29 cells were maintained at a concentration of 1×10^6^ cells/mL by diluting every 2–3 days in fresh medium to maintain exponential cell growth. HT-29 cells were rinsed with phosphate buffered saline (PBS), trypsinized, counted, and seeded at a density of 2×10^5^ cells/mL onto 96-well plates, with 100 µL per well. After overnight attachment, cells were treated during 48 and 72 h in triplicate with different reagents, including rShh, cyclopamine, purmorphamine, butyrate, IFN-γ, EGF, or LPS. BrdU was added to wells 4 h prior to the plate reading at a wavelength of 450 nm.

For assessing cell viability, experiments with the same settings were performed in parallel, and the labeling reagent 3-(4,5-dimethylthiazol-2-yl)-2,5-diphenyltetrazolium bromide (MTT) (Sigma-Aldrich, St. Louis, MO, USA) was added to wells, and the plates were read at a wavelength of 595 nm. The MTT test serves as an indirect marker for proliferation and cell viability by measuring the mitochondrial activity of cells.

### Assessment of Apoptosis

HT-29 cells were plated at a density of 10^5^ cells/well in six-well plates. After overnight attachment, cells were treated with different reagents, including the anti-CD95/APO-1 monoclonal antibody (Southern Biotech, Birmingham, AL, USA) to induce apoptosis, or vehicle control (DMSO, 0.2%), in triplicate, for 24 hr, followed by washing with PBS, trypsinization, and centrifugation. For the assessment of cell viability and both early and late-stage apoptosis, cells were stained with the Annexin-V kit and the 7-AAD Kit (both from e-Biosciences, San Diego, CA, USA). Briefly, cells were washed twice in cold PBS, resuspended in binding buffer (1×10^6^ cells in 0.1 ml), and 10 µl of FITC-conjugated Annexin-V and 20 µL of 7-AAD were added. Cells were incubated for 15 min in the dark, an additional 400 µL of binding buffer were added, and the cells were analyzed within 1 hour by flow cytometry. Acquisition was performed on a FACSCalibur using the CellQuest Pro software (BD Biosciences, San Jose, CA, USA), and quantitatively analyzed using the Winlist software program (Verity Software House, Topsham, ME, USA). Each analysis was performed on at least 30,000 events.

### Statistical Analysis

Statistical analyses were performed using the statistical software SPSS for Windows (Version 10.1, SPSS Inc., 1989–1999, USA). Statistical differences among the experimental groups were evaluated with the one-way ANOVA test in which pair wise multiple comparisons were carried out using the Dunnett’s T3 test. The level of significance was set at p<0.05.

## Results

### Expression and Modulation of Hh Pathway Components in HT-29 Cells

**Figure 1:**
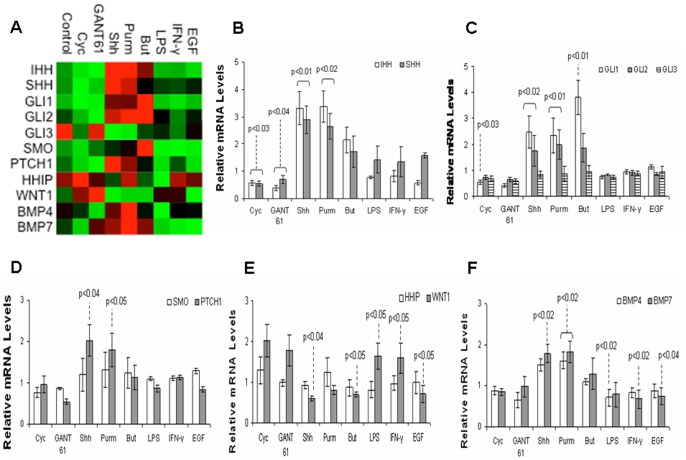
Gene modulation in HT-29 cells upon exposure to different stimuli. Heat map representation of gene expression determined by RT-qPCR. HT-29 cells were treated with Shh (Sonic Hedgehog), Purm (purmorphamine), But (butyrate), LPS, IFN-γ, EGF, with or without the addition of cyclopamine (Cyc), GANT 61, or DMSO (vehicle) for 24 hours (A). Histograms of individual gene expression fold changes in HT-29 cells: IHH and SHH (B); GLI1, GLI2 and GLI3 (C); SMO and PTCH1 (D); HHIP and WNT1 (E); and BMP4 and BMP7 (F). The graphs use the same samples as in (A). Values represent the means ± SEM of three independent experiments and are normalized to GAPDH, beta-actin, and RPL32 RNA genes. Significant changes in relation to the control group are highlighted.

We investigated the effect of directly stimulating or inhibiting the Hh pathway, and the exposure to bacterial products (LPS), cytokine (IFN-γ), and growth-factor (EGF), in the gene expression of HT-29 cells by quantitative real-time PCR analyses. Several genes directly involved in the Hh pathway and others which possibly interact with Hh signaling were profiled (Figure 1A). All genes studied were expressed as fold changes in relation to the control group (arbitrarily normalized to 1).

Levels of *IHH* and *SHH* mRNA significantly increased after exposure to rShh-peptide or to the Hh agonist purmorphamine (*P*<0.02), and decreased after treating HT-29 cells with the Hh antagonists cyclopamine (*P*<0.03) or GANT 61 (*P*<0.04) (Figure 1B).

In regard to the downstream transcription factors of the Hh pathway, treatment with rShh-peptide or purmorphamine significantly increased the HT-29 levels of *GLI-1* and *GLI-2* mRNA (*P*<0.02), the main activators of Hh target genes and indicators of the Hh pathway activation [23]. Levels of *GLI-1* mRNA also increased after treatment with the short chain fatty acid butyrate (*P*<0.01), but decreased upon exposure to cyclopamine (*P*<0.03). Levels of *GLI-3* did not change significantly, irrespective of the stimuli (Figure 1C).

In regard to the transmembrane receptors Patched (Ptch) and Smoothened (Smo), we observed that the expression of *PTCH1* increased upon exposure to rShh-peptide or purmorphamine (*P*<0.05). Levels of *SMO* did not change significantly in HT-29 cells independently of the treatment (Figure 1D).

The expression of the inhibitor *HHIP* did not change significantly, irrespective of the stimuli. The expression of *WNT1*, component of the Wnt signaling pathway significantly decreased after butyrate, rShh-peptide, or EGF (*P*<0.05), while it increased upon exposure to LPS or IFN-γ (*P*<0.05). (Figure 1E).

We assessed the levels of mRNA for BMP4, known to be present in intestinal epithelial cells and a target of both Hh [24] and Wnt [11] pathways, and BMP7, also expressed in the colon but to a lesser extent within the epithelium [25]. The expression of *BMP4* significantly decreased after the treatment with LPS (*P*<0.02), while the expression of *BMP7* significantly decreased after IFN-γ (*P*<0.02) or EGF (*P*<0.04). Levels of *BMP7* significantly increased following rShh-peptide (*P*<0.02), while both *BMP4* and *BMP7* increased after purmorphamine (*P*<0.02) (Figure 1F). Results on the expression and modulation of Hh pathway in other colon cancer cells indicate a similar response in HCT8 and HCT116, but not in Caco-2 cells (Figure S1).

### Subcellular Levels and Distribution of Gli-1 and β-catenin in HT-29 Cells

**Figure 2:**
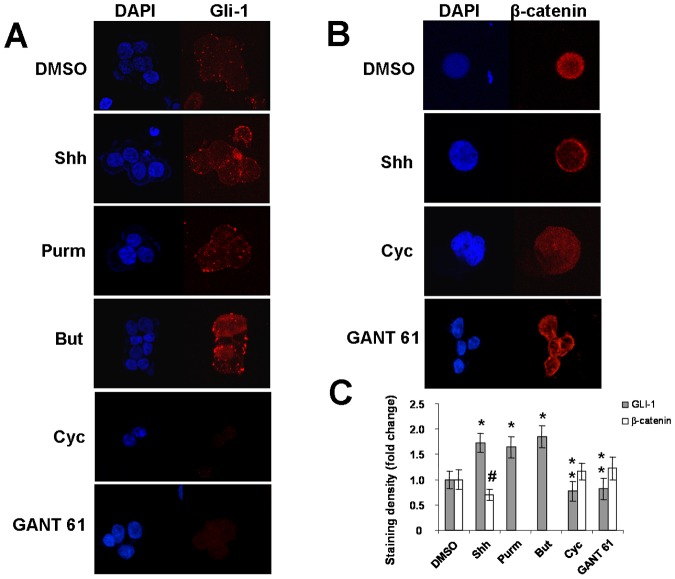
Distribution and levels of Gli-1 and β-catenin in HT-29 cells. Confocal microscopy of cytospin preparations showing the relative nuclear and cytoplasmic distribution and levels of Gli-1 (A) and β-catenin (B) in HT-29 cells exposed to different stimuli for 24 hours. Analysis of protein staining densities confirm that Gli-1 significantly increases after treatment with Shh (Sonic Hedgehog), Purm (purmorphamine), or But (butyrate), compared to cells treated with DMSO (vehicle) (*P<0.04), and compared to cells treated with Cyc (cyclopamine) or GANT 61 (**P<0.02). Density of β-catenin significantly decreases upon treatment with Shh (# *P*<0.04) compared to DMSO, Cyc, or GANT 61 (C). Nuclei are stained with DAPI (blue). Micrograph panel is representative of 3–4 experiments for each condition (Original magnification ×1000).

To further confirm these findings, we next investigated the expression of the Gli-1 protein, transcription factor of the Hh pathway in HT-29 cells. Low levels of Gli-1 were detected, predominantly in the cytosol. When HT-29 cells were exposed to rShh-peptide, purmorphamine, or butyrate, levels of Gli-1 increased with a marked relative subcellular distribution into the nucleus. An opposing effect was shown with the addition of the Hh antagonist cyclopamine (Figure 2A). Because Gli-1 is one of the indicators of the Hh pathway activation, enhanced expression and nuclear translocation indicate that Hh pathway is active and can be regulated in HT-29 cells. Considering that Hh signaling is known to negatively regulate Wnt signaling in the colonic epithelium [11], we next studied possible interactions between Wnt signaling and the Hh expression or activity in HT-29 cells. We detected a constitutive expression of β-catenin, an integral component of the Wnt pathway, predominantly in the nucleus of HT-29 cells. Exposure of these cells to rShh-peptide during 24 hours reduced the expression of β-catenin and restricted it to its membrane-bound form (Figure 2B). Densitometric analysis of staining densities confirmed that levels of Gli-1 were significantly higher after exposure to rShh-peptide, purmorphamine, or butyrate (*P<0.04), and significantly lower after cyclopamine or GANT 61 (**P<0.02), compared to vehicle treated cells. In regard to β-catenin staining, densities became significantly lower after exposure to rShh-peptide (#*P*<0.04) compared to vehicle, cyclopamine or GANT 61 treated cells (Figure 2C). Similar staining patterns for Gli-1 and β-catenin were observed in HCT8 and HCT116 cells, but not in Caco-2 cells (Figures S2, S3, and S4).

### Effect of Hh Pathway on the Inflammatory Activity of HT-29 Cells

**Figure 3:**
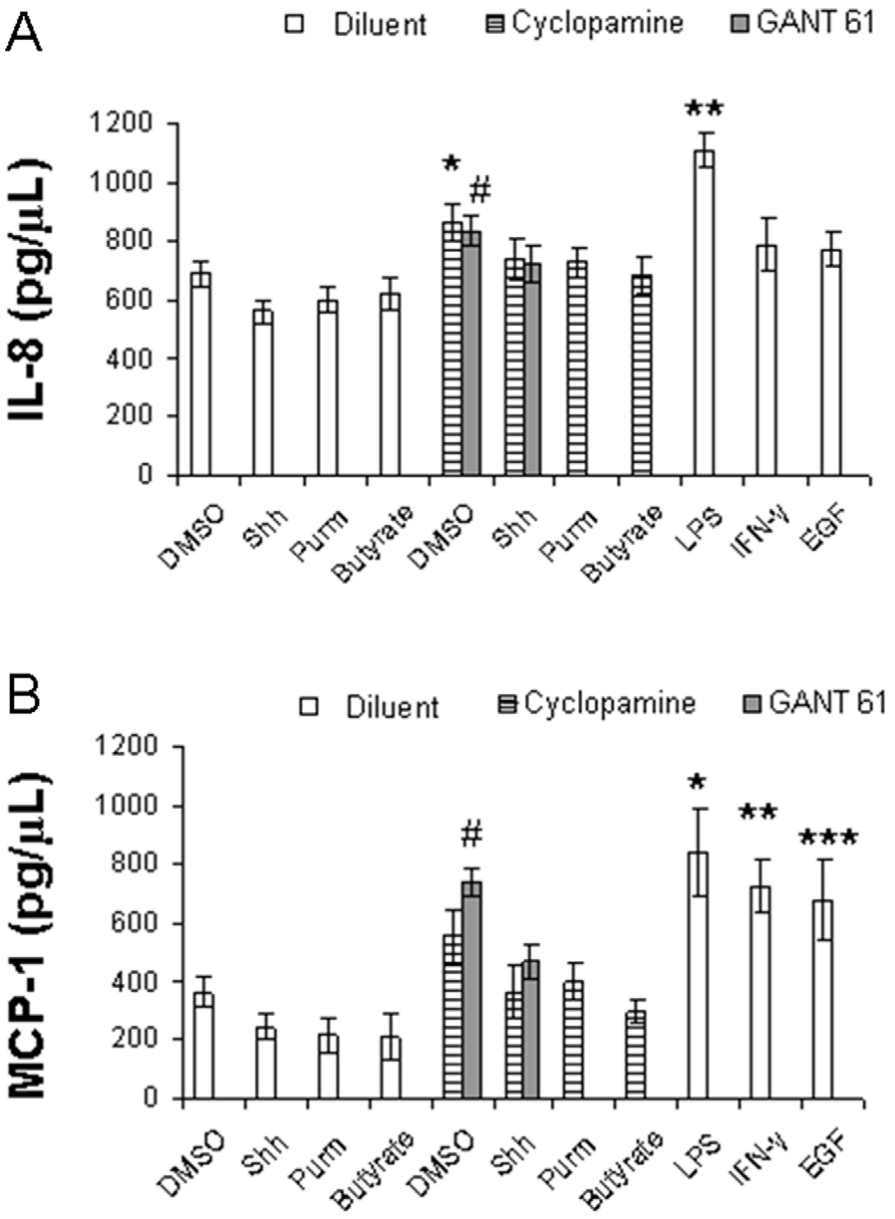
Production of cytokines by HT-29 cell cultures upon exposure to different stimuli. Concentration of cytokines in supernatants of 24 hour cultures of HT-29 cells measured by ELISA. Histograms show the levels of IL-8 (A) and MCP-1 (B), under various conditions: treatment with Shh (Sonic Hedgehog), Purm (purmorphamine), Butyrate, LPS, IFN-γ, EGF, with or without the addition of cyclopamine, GANT 61, or DMSO (vehicle control) for 24 hours. IL-8 levels are significantly lower in cells treated with either Shh, purmorphamine, or butyrate compared to those treated with cyclopamine (^*^P<0.02), GANT 61 (*^#^P*<0.04), or LPS (^**^P<0.03), respectively (A). MCP-1 levels are significantly lower in cells treated with Shh, purmorphamine, or butyrate compared to the ones treated with GANT 61 (#*P*<0.02), LPS (^*^P<0.03) IFN-γ (^**^P<0.03), or EGF (^***^P<0.04), respectively (B). Data are expressed as the mean ± SEM of 6 independent experiments.

To elucidate the functional effects of Hh pathway on HT-29 cells, we first measured the concentration of interleukin-8 (IL-8) and monocyte chemoattractant protein-1 (MCP-1), inflammatory mediators known to be produced by epithelial cells and to act mainly as chemoattractant molecules to leukocytes [26]. The levels of IL-8 were significantly lower in HT-29 cells treated with either rShh-peptide, purmorphamine, or butyrate compared to those treated with cyclopamine (*P<0.02), GANT 61 (#*P*<0.04), or LPS (**P<0.03), respectively (Figure 3A). MCP-1 production decreased significantly in cells treated with rShh-peptide, purmorphamine, or butyrate compared to the ones exposed to GANT 61 (#*P*<0.02), LPS (*P<0.03), IFN-γ (**P<0.03), or EGF (***P<0.04), respectively (Figure 3B). Data are expressed as the mean ± SEM of 6 independent experiments. In parallel experiments on IL-8 and MCP-1 production, HCT8 and HCT116 cells were shown to respond similarly to HT-29 cells, in contrast to Caco-2 cells (Figures S5 and S6).

### Effect of Hh Pathway on Chemotactic Response of Monocytes to HT-29 Cell Culture Supernatants

**Figure 4:**
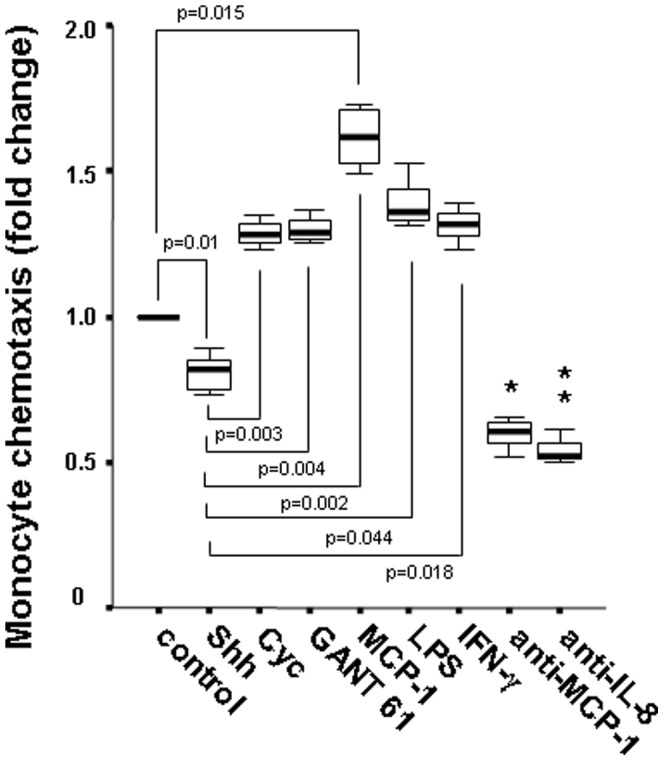
Monocyte chemotaxis induced by cell culture supernatants of HT-29 cells upon exposure to different stimuli. Monocytes were incubated in the upper chambers of a 5 µm-pore-size polycarbonate filter in a transwell system, with the 24 h-culture supernatants of HT-29 cells treated with rShh, cyclopamine, LPS, or IFN-γ in the lower chambers. Supernatants of HT-29 untreated cells were used as controls and values were arbitrarily normalized to 1. Additional controls were obtained by the incubation with the chemoattractant MCP-1, and anti-MCP-1 antibody, as positive and negative controls, respectively. *Cell migration is significantly lower in cells treated with Shh compared to controls (*P = *0.01), and those treated with GANT61 (*P = *0.005), cyclopamine (*P = *0.003), LPS (*P = *0.042), IFN-γ (*P = *0.017), and MCP-1 (*P = *0.001), respectively. **Anti-MCP-1 significantly decreased chemokinesis compared with GANT61, cyclopamine, LPS, IFN-γ, or MCP-1 (*P*<0.02),. Data are expressed as the mean ± SEM of 3 independent experiments.

Next, supernatants of HT-29 cells exposed to different stimuli were analyzed for the ability to induce monocyte chemotaxis in a transwell system. Monocyte migration significantly decreased using superantants of HT-29 cells treated with rShh compared to cyclopamine (*P* = 0.003), GANT61 (*P* = 0.004), LPS (*P* = 0.044), IFN-γ (*P* = 0.018), and the chemoattractant MCP-1 (*P* = 0.002). Antibody against MCP-1 was utilized as an additional assay control (Figure 4).

### Effect of Hh Pathway on Survival and Proliferative Activity of HT-29 Cells

**Figure 5:**
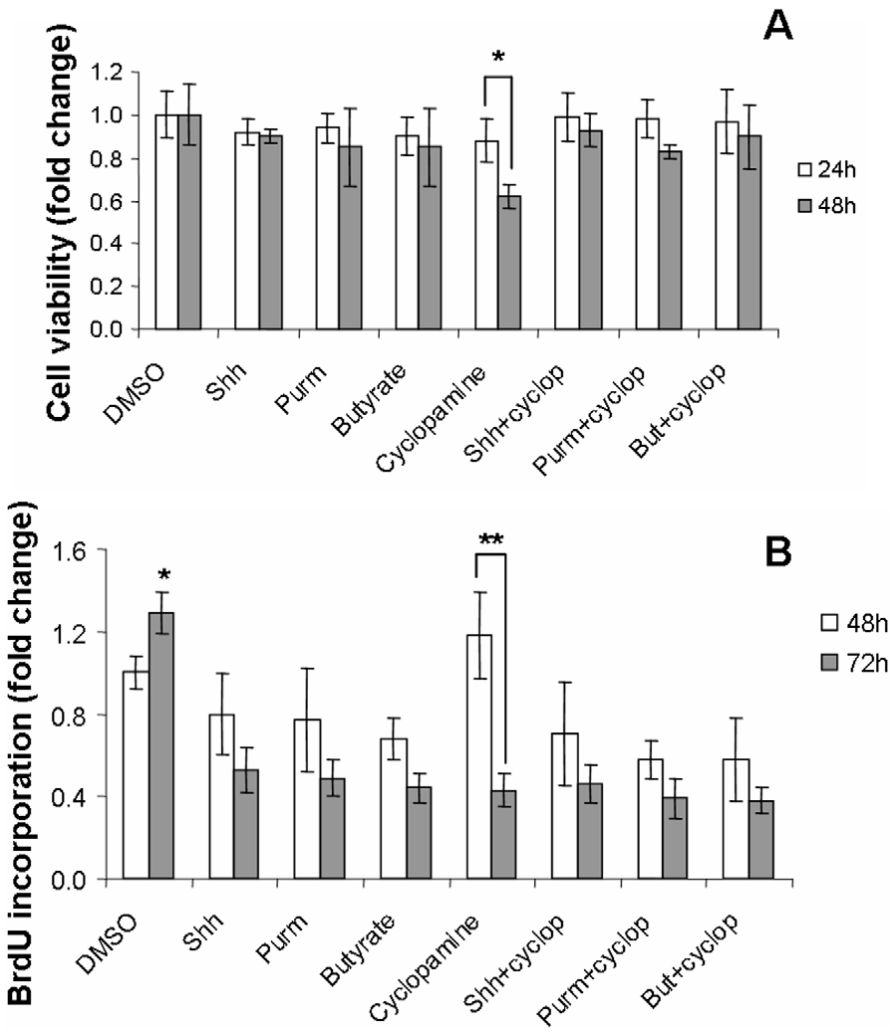
Survival and proliferative activity of HT-29 cells upon exposure to different stimuli. HT-29 cell viability was analyzed at different time points with different treatments, using the MTT assay. When HT-29 cells were exposed to cyclopamine, cell viability decreased significantly comparing 48 with 24 hours (^*^P<0.05) (A). For the purpose of analyzing changes in the proliferative activity of HT-29 cells upon different treatments, the cellular incorporation of BrdU was measured at 48 and 72 hours. After 72 hours, BrdU incorporation was maintained in vehicle-treated cells (DMSO), while it significantly decreased in all other treatment groups (^*^P<0.04). Within the group of cyclopamine exposed HT-29 cells, BrdU incorporation decreased significantly from 48 to 72 hours (^**^P<0.05) (B). Data are expressed as the mean ± SEM of 3 independent experiments.

HT-29 cell viability was analyzed at different time points with different treatments, using the MTT assay. Although no difference was found among HT-29 cell treatments in the first 24 hours, cell viability decreased significantly at 48 hours when cells were exposed to cyclopamine (*P<0.04) (Figure 5A).

For the purpose of analyzing changes in the proliferative activity of HT-29 cells upon different treatments, the cellular incorporation of BrdU was measured at 48 and 72 hours. Although no significant difference among the treatment groups was detected in the first 48 hours, we identified a tendency for increase in BrdU incorporation in the cyclopamine treated cells. However, a significant decrease in BrdU incorporation was found within the group of cyclopamine exposed HT-29 cells, from 48 to 72 hours (**P<0.05). After 72 hours, BrdU incorporation was maintained in vehicle-treated cells (DMSO), while it became significantly lower in all other treatment groups (*P<0.05) (Figure 5B). All data in both the MTT and the BrdU assays are expressed as the mean ± SEM of 3 independent experiments each. Studies with other colon cancer cells showed a similar response in HCT8 and HCT116 cells, but not in Caco-2 (Figures S7 and S8).

### Effect of Hh Pathway on CD95-mediated Apoptosis in HT-29 Cells

**Figure 6:**
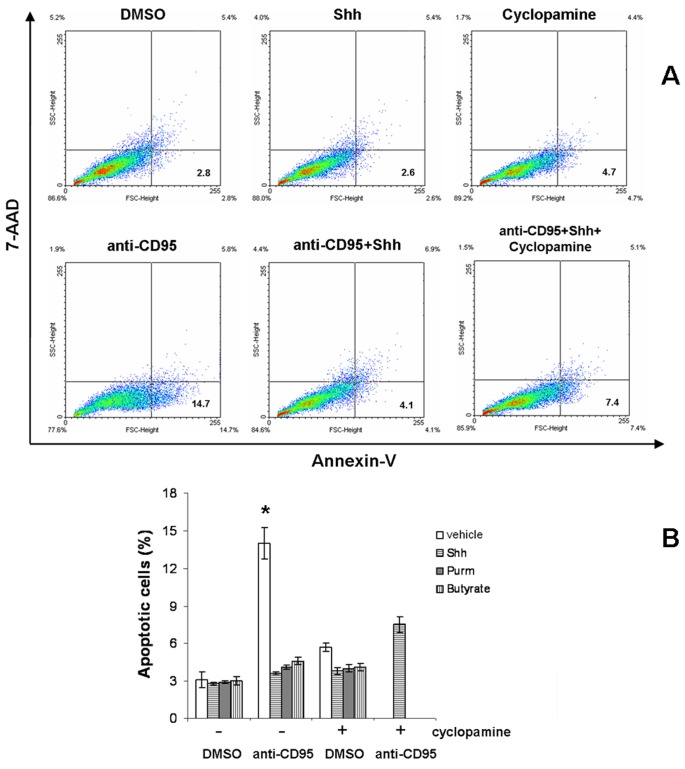
Relationship between apoptosis and the activity of Hedgehog pathway in HT-29 cells. Flow cytometric demonstration of HT-29 apoptosis after 24 hours of exposure to different stimuli, as assessed by annexin-V/7-AAD. Left lower quadrant indicates double negative cells while right lower quadrant indicates annexin-V-positive cells (A). Treatment with Shh, purmorphamine or butyrate significantly abrogates the anti-CD95 induced apoptosis (^*^P<0.02), which is partially restored by the addition of cyclopamine (B). Data are expressed as the mean ± SEM of 3 independent experiments.

Flow cytometric demonstration of HT-29 apoptosis after 24 hours of exposure to different stimuli, as assessed by annexin-V/7-AAD. Left lower quadrant indicates double negative cells, while right lower quadrant indicates early apoptotic annexin-V-positive cells, and the upper quadrants indicate late apoptotic or necrotic cells (Figure 6A). Treatment with rShh-peptide, purmorphamine or butyrate significantly abrogates the anti-CD95 (anti-Fas) induced apoptosis (*P<0.01), which is partially restored by the addition of cyclopamine (Figure 6B). Data are expressed as the mean ± SEM of 3 independent experiments. A protective effect of rShh-peptide against apoptosis was also observed in HCT8 cells, but not in Caco-2 cells (Figures S9 and S10).

### HCT8, HCT116, and Caco-2 Human Colon Cancer Cell Lines

All results obtained with these cells are presented in the Supporting Information S1 session.

## Discussion

In this study, we show that the epithelial HT-29 human colon carcinoma cell line expresses Hh pathway components which can be modulated by different exogenous stimuli. In addition, we demonstrate that the biological role of the Hh pathway in HT-29 cells ranges from the control of cell survival to the regulation of cytokine production. The Hh pathway has been broadly detected in fetal tissues and cells, whereas in the adult gastrointestinal tract Hh elements have been predominantly found in the epithelial lining under physiological conditions [11], [27]. Nevertheless, the Hh pathway seems to be reactivated in several cancers [14], [28], in which the presence of Hh components suggests the participation of this pathway in mechanisms regulating either cell proliferation or apoptosis [29]. Therefore, we were interested in investigating whether Hh signaling would play a role in the homeostatic control of HT-29 cells survival and function.

HT-29 cells constitute an epithelial colonic cancer cell line that has been utilized as a model for studying epithelial cells, and also cancer pathways and for the development of new therapeutic approaches. In this study, we analyzed the baseline levels and the potential modulation of Hh pathway components in HT-29 cells upon exposure to different exogenous stimuli. The Hh signaling pathway has been shown to actively participate in the initiation and progression of gastrointestinal tumors [13], [14], [21]. Nevertheless, overexpression of Hh signaling molecules has also been reported in colon cancer [20], [30]. In the present study, the expression of Hh components in HT-29 colon cancer cell line was demonstrated both at the mRNA and protein levels.

The expression and modulation of a selection of Hh pathway-related genes and other indirectly associated genes were investigated by quantitative real-time PCR in HT-29 cells. We demonstrated the presence of *IHH*, *SHH*, the main indicators of the Hh pathway activation *GLI-1* and *GLI-2*, and of *PTCH1*, and the ability to modulate mRNA levels of those genes following treatment of HT-29 cells with the Hh antagonist cyclopamine or the specific stimulation with rShh-peptide or the Hh agonist purmorphamine. Similar to another study, we also observed *GLI-1* mRNA upregulation after treatment with the short chain fatty acid butyrate [11], while levels decreased after inhibition with cyclopamine. Of note, exposure to bacterial products, inflammatory cytokine, or growth factor, had minimal or no impact on the Hh pathway gene modulation in HT-29 cells. However, when investigating epithelial bone morphogenetic proteins (Bmps) we observed a marked upregulation of *BMP4* and *BMP7* following Hh stimulation, but the levels of *BMP7* decreased after IFN-γ or EGF, while levels of *BMP4* decreased with the addition of LPS to HT-29 cell cultures, probably indicating a downregulation in response to inflammatory signals. Bmps are morphogens of the transforming growth factor β superfamily, which were shown to play a critical role in epithelial proliferation and terminal differentiation and maturation of cells from the secretory lineage of the intestine [31]. Moreover, Bmps are known targets of the Hh pathway during development [32], and *BMP4* and *BMP7* were identified as potential mediators of the mesenchymal-epithelial interaction in response to Hh signaling within the mouse intestine [24]. Therefore, here we further confirm the expression of *Bmps* and the modulation by Hh signaling in differentiated human colonic HT-29 cells. The expression of *WNT1*, an antagonist of the Hh pathway in epithelial cells markedly decreased after exposure to rShh-peptide and butyrate, while it increased upon exposure to IFN-γ or LPS. Components of the Wnt/β-catenin pathway are known to be overexpressed in most colon cancers and in HT-29 cells their presence supports a deregulated hyperproliferative status, which appears to be counteracted by the Hh signaling casacade in a paracrine and autocrine fashion [33]. Response of HT-29 cells to IFN-γ and LPS may indicate epigenetic modulation of the Wnt pathway by proinflammatory stimuli, and appear to unveil a complex interaction of multiple genes and converging signaling pathways involved in physiologic processes, but also in inflammation and oncogenesis of colonic epithelial cells.

In HT-29 cells, the baseline expression of Gli1 protein was low but consistently increased both in the cytoplasm and nucleus, following exposure to Shh peptide, the Hh agonist purmorphamine, and the short chain fatty acid butyrate, while levels decreased and disappeared from the nucleus after treatment with Hh inhibitors. This is in contrast with the high constitutive levels observed in gastric [15] and breast [34] carcinomas, but still implies that Hh activation can be modulated by exogenous stimuli in HT-29 cells and other colon cancer cells. In parallel, we demonstrated a low constitutive expression of β-catenin, member of the Wnt pathway in HT-29 cells. Interestingly, we additionally observed an opposing effect of the same exogenous stimuli on β-catenin compared to Gli-1, in terms of protein subcellular levels and distribution in HT-29 cells. It is well-established that deregulation of the β-catenin/Wnt pathway components leads to the nuclear accumulation of β-catenin and to tumor formation [35]. However, β-catenin transactivation is not exclusively related to activating mutations of the Wnt pathway components [36], and it was also shown to be induced by proinflammatory signals [37]. In accordance to previous studies suggesting the existence of interactions between Wnt signaling and Hh activity in the colonic epithelium [11], [38], our findings appear to support the suggestion that Hh signaling would possibly have the potential to negatively regulate the Wnt pathway in HT-29 cells and other colon cancer cell lines. In particular, exposure to Hh inhibitors resulted in the nuclear accumulation, while Hh activation led to β-catenin reduction and to a predominant peripheral cytoplasmic localization.

In addition to comprise a surface for absorption and a mechanical barrier separating the external environment from the internal milieu, colonic epithelial cells are also involved in inflammatory processes, and have been reported to be a source of chemokines [39], [40]. In this study, we demonstrated that the levels of IL-8, a potent neutrophil chemotactic and activating agent of the CXC subfamily of chemokines [26], decreased following Hh pathway activation, in contrast to Hh blockade or to LPS induction. Similarly, MCP-1, of the CC subfamily with chemotactic activity to monocytes and other immune cells [41], decreased following Hh activation, in opposition to effects induced by GANT 61, LPS, IFN-γ, or EGF. Our findings in HT-29 cells and other colon cancer cells appear to corroborate the importance of chemokines as critical attractors for migrating or invading malignant cells toward the blood vessels [42], and to control migration of metastatic cells to distant organs [43]. Furthermore, chemokines downregulation by Hh signaling may constitute a mechanism to circumvent immune surveillance in colon cancer cells, with potential implications in the severity and the metastatic potential of colorectal cancer.

In the context of Hh signaling data on colorectal cancer [14], [19], [28], and cancer cell lines [11], [17], [22] have been controversial. In contrast to our results, Yauch et al. (2008) for example, did not detect inhibition of epithelial cells by Hh pathway blockade and proposed that Hh signaling acts only on the tumor stromal cells [44]. The explanation for these discrepancies is unknown, but it has been proposed that they might be due, at least in part, to distinct methodologies, analysis of unparalleled pathological samples, the consequences of epigenetic modifications to gene expression, and also to different response kinetics of stromal versus epithelial cells. On the other hand, similar to our findings, recent reports have indicated that Hh signaling is required for colon cancer cell survival, and that blocking the Hh pathway with cyclopamine [30] or shRNA, induced apoptosis [17]. Other recent studies confirmed the relevance of Hh signaling to cellular survival through the activation of *GLI-1* and *GLI-2* in human colon carcinoma cells using GANT 61, an inhibitor of the Hh pathway by targeting Gli [45], [46]. Moreover, recent clinical data also demonstrated that the increasing expression of Hh pathway components are indicators for a poor prognosis in patients with colon cancer. [47].

In this study, we identified an early tendency toward BrdU incorporation, followed by a late decrease in BrdU uptake after Hh inhibition. In a complementary experiment, we demonstrated the reduction in HT-29 and HCT116 cell viability also following Hh pathway inhibition with cyclopamine, in accordance to a previous study [48]. To further understand the mechanistic link between Hh pathway and the fate of colon cancer cells, we stimulated cells via the Fas/FasL system in order to mediate cell death by apoptosis. Similar to other studies [30], we demonstrated that Hh agonists are able to attenuate apoptosis in both HT-29 and HCT8, human colon cancer cell lines. Therefore, in contrast to another study [21], we propose that Hh signaling may not be critical for the proliferation of colon cancer cells with high differentiation status such as HT-29, HCT8 and HCT116 cells. Nevertheless, our results suggest that viability may be, at least in part, dependent on the Hh pathway, further supporting the concept that active Hh signaling in the colon cancer cells is likely to be involved in cell differentiation and also the protection against apoptosis. On the other hand, the low expression and activity of Hh pathway is consistent with the lack of response to Hh agonists or blockers in Caco-2 cells and, in addition to low levels of Wnt/β-catenin it may be explained by temporal patterns of gene expression during cell–cell adhesion-initiated polarization of cultured Caco-2 cells [49].

In conclusion, we show that the Hh pathway is a key controller of colon cancer cells function, as demonstrated by its effect in promoting dampening of inflammatory signals and antagonizing apoptosis. The Hh activity in colonic epithelial cells has probable homeostatic implications for the development of colonic inflammation and malignancies, since the differential expression of Hh components may underlie abnormalities in the local immune response and in epithelial barrier integrity. Taken together, these results highlight the importance of Hh activation as a potential therapeutic target in models of human colorectal cancer.

## Supporting Information

Figure S1 **Gene modulation in HCT8, HCT116, and Caco-2 cells upon exposure to rShh, cyclopamine, or DMSO (vehicle) for 24 hours, determined by RT-qPCR.** Histograms of individual cell lines express fold changes of: *IHH*, *SHH*, *GLI1*, *GLI2*, *GLI3*, *SMO*, *PTCH1*, *HHIP*, *WNT1*, *BMP4* and *BMP7*. Values represent the means ± SEM of three independent experiments and are normalized to GAPDH, beta-actin, and RPL32 RNA genes. Significant changes in relation to the control group are highlighted.
(TIF)

Figure S2 **Distribution and levels of Gli-1 and β-catenin in HCT8 cells.** Relative nuclear and cytoplasmic distribution and levels of Gli-1 (left panel, A) and β-catenin (right panel, B) in HCT8 cells exposed to different stimuli for 24 hours were analyzed by confocal microscopy. In HCT8 cell, staining densities indicate that Gli-1 protein increases after treatment with Shh (Sonic Hedgehog), or Purm (purmorphamine), compared to cells treated with DMSO (vehicle), or Cyc (cyclopamine). Density of β-catenin decreases upon treatment with Shh compared to either DMSO or Cyc. Nuclei are stained with DAPI (blue). Micrograph panel is representative of 3 experiments for each condition (Original magnification ×1000).
(TIF)

Figure S3 **Distribution and levels of Gli-1 and β-catenin in HCT116 cells.** Relative nuclear and cytoplasmic distribution and levels of Gli-1 (left panel, A) and β-catenin (right panel, B) in HCT116 cells exposed to different stimuli for 24 hours were analyzed by confocal microscopy. In HCT116, staining densities show that Gli-1 protein increases after treatment with Shh (Sonic Hedgehog), or Purm (purmorphamine), compared to cells treated with DMSO (vehicle), or Cyc (cyclopamine). Density of β-catenin decreases upon treatment with Shh compared to either DMSO or Cyc. Nuclei are stained with DAPI (blue). Micrograph panel is representative of 3 experiments for each condition (Original magnification ×1000).
(TIF)

Figure S4 **Distribution and levels of Gli-1 and β-catenin in Caco-2 cells.** Relative nuclear and cytoplasmic distribution and levels of Gli-1 (left panel, A) and β-catenin (right panel, B) in Caco-2 cells exposed to different stimuli for 24 hours were analyzed by confocal microscopy. In Caco-2 cells, staining of Gli-1 protein is almost undetectable and do not change after treatment with Shh (Sonic Hedgehog), Purm (purmorphamine), Cyc (cyclopamine), compared to cells treated with DMSO (vehicle). Densities of β-catenin are low and do not change upon treatment with Shh or Cyc, compared to DMSO. Nuclei are stained with DAPI (blue). Micrograph panel is representative of 3 experiments for each condition (Original magnification ×1000).
(TIF)

Figure S5 **Production of interleukin-8 (IL-8) by HCT8, HCT116, and Caco-2 cell cultures upon exposure to different stimuli.** Concentration of IL-8 in supernatants of 24 hour cultures was measured by ELISA. Histograms show the levels of IL-8, under various conditions: treatment with Shh (Sonic Hedgehog), Purm (purmorphamine), Butyrate, LPS, IFN-γ, EGF, with or without the addition of cyclopamine, or DMSO (vehicle control) for 24 hours. In HCT116 cells, IL-8 levels are significantly lower in cells treated with either Shh, purmorphamine, or butyrate compared to those treated with cyclopamine (*P<0.04). In HCT8 and Caco-2 cells, levels did not change significantly. Data are expressed as the mean ± SEM of 3 independent experiments
(TIF)

Figure S6 **Production of monocyte chemotactic protein 1 (MCP-1) by HCT8, HCT116, and Caco-2 cell cultures upon exposure to different stimuli.** Concentration of MCP-1 in supernatants of 24 hour cultures was measured by ELISA. Histograms show the levels of MCP-1, under various conditions: treatment with Shh (Sonic Hedgehog), Purm (purmorphamine), Butyrate, LPS, IFN-γ, EGF, with or without the addition of cyclopamine, or DMSO (vehicle control) for 24 hours. In HCT116 cells, MCP-1 production decreased significantly after treatment with rShh-peptide, purmorphamine, or butyrate compared to the ones exposed cyclopamine (*P = 0.037), to LPS (**P = 0.043), or IFN-γ (***P<0.049), respectively. In HCT8 cells, levels of MCP-1 were significantly lower following exposure to either rShh-peptide, purmorphamine, or butyrate compared to those treated with cyclopamine (*P<0.04). Data are expressed as the mean ± SEM of 3 independent experiments.
(TIF)

Figure S7 **Survival of HCT8, HCT116, and Caco-2 cells upon exposure to different stimuli.** Cell viability was analyzed at different time points with different treatments, using the MTT assay. When HCT116 cells were exposed to cyclopamine, cell viability decreased significantly comparing 48 with 24 hours (*P<0.04). Data are expressed as the mean ± SEM of 3 independent experiments each.
(TIF)

Figure S8 **Proliferative activity of HCT8, HCT116, and Caco-2 cells upon exposure to different stimuli.** Changes in the proliferative activity of cells were analyzed with different treatments, using the cellular incorporation of BrdU, measured at 48 and 72 hours. A significant decrease in BrdU incorporation was observed from 48 to 72 hours within the group of cyclopamine exposed HCT8 (*P*<0.04) and HCT116 (*P*<0.043) cells, respectively. Data are expressed as the mean ± SEM of 3 independent experiments each.
(TIF)

Figure S9 **Relationship between apoptosis and the activity of Hedgehog pathway in HCT8 cells.** Flow cytometric demonstration of cell apoptosis after 24 hours of exposure to different stimuli, as assessed by annexin-V/7-AAD. Left lower quadrant indicates double negative cells while right lower quadrant indicates annexin-V-positive cells (A). For HCT8 cells, treatment with rShh significantly inhibited the anti-CD95 induced apoptosis (*P*<0.049), which is partially restored by the addition of cyclopamine (B). Data are expressed as the mean ± SEM of 3 independent experiments.
(TIF)

Figure S10 **Relationship between apoptosis and the activity of Hedgehog pathway in Caco-2 cells.** Flow cytometric demonstration of cell apoptosis after 24 hours of exposure to different stimuli, as assessed by annexin-V/7-AAD. Left lower quadrant indicates double negative cells while right lower quadrant indicates annexin-V-positive cells (A). For Caco-2 cells, anti-CD95 mediated cell death was not significantly modified by rShh or cyclopamine (B). Data are expressed as the mean ± SEM of 3 independent experiments.
(TIF)

Supporting Information S1 **Supporting Information.**
(DOC)
