# Supplementary figures and images for: Hedgehog Pathway Signaling Regulates Human Colon Carcinoma HT-29 Epithelial Cell Line Apoptosis and Cytokine Secretion
Source: PLoS One. 2012 Sep 19;7(9):e45332. doi: 10.1371/journal.pone.0045332 (PMC3446889; doi:10.1371/journal.pone.0045332)

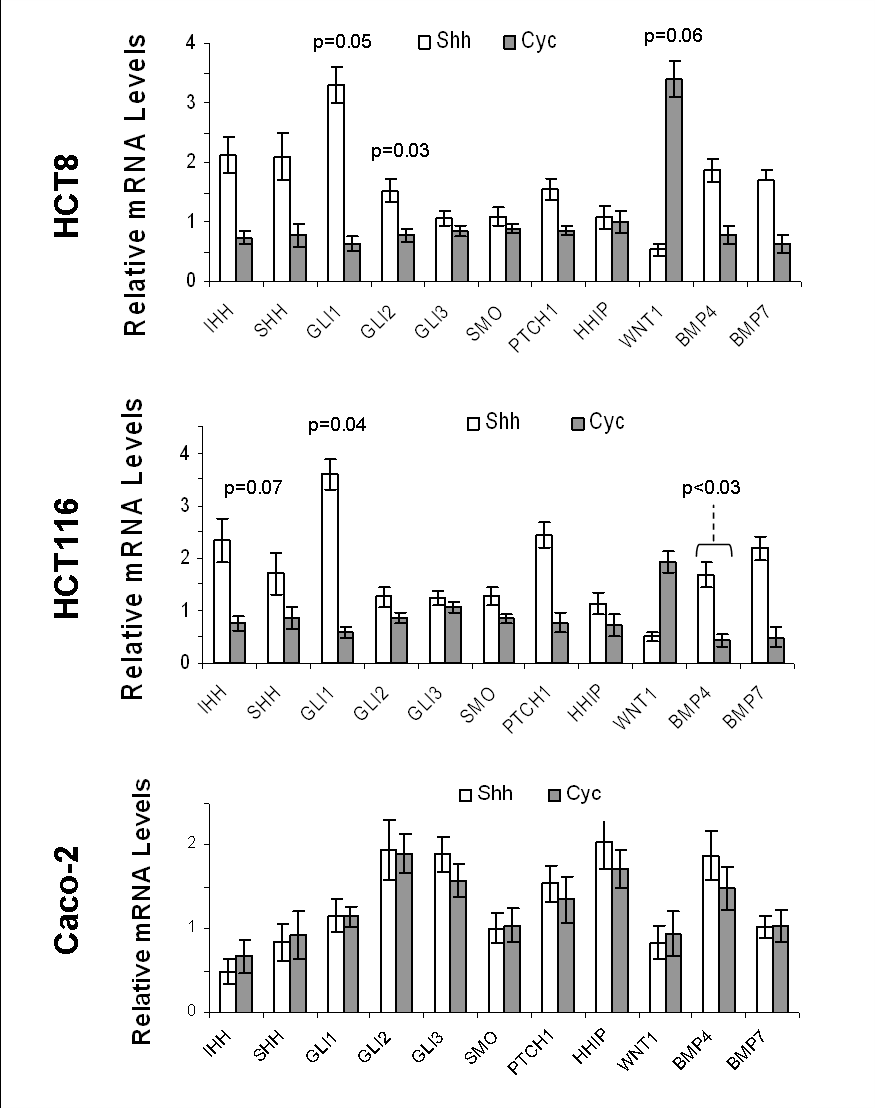

Supplement: Figure S1 — Gene modulation in HCT8, HCT116, and Caco-2 cells upon exposure to rShh, cyclopamine, or DMSO (vehicle) for 24 hours, determined by RT-qPCR. Histograms of individual cell lines express fold changes of: IHH, SHH, GLI1, GLI2, GLI3, SMO, PTCH1, HHIP, WNT1, BMP4 and BMP7. Values represent the means ± SEM of three independent experiments and are normalized to GAPDH, beta-actin, and RPL32 RNA genes. Significant changes in relation to the control group are highlighted. (TIF) [file pone.0045332.s001.tif]

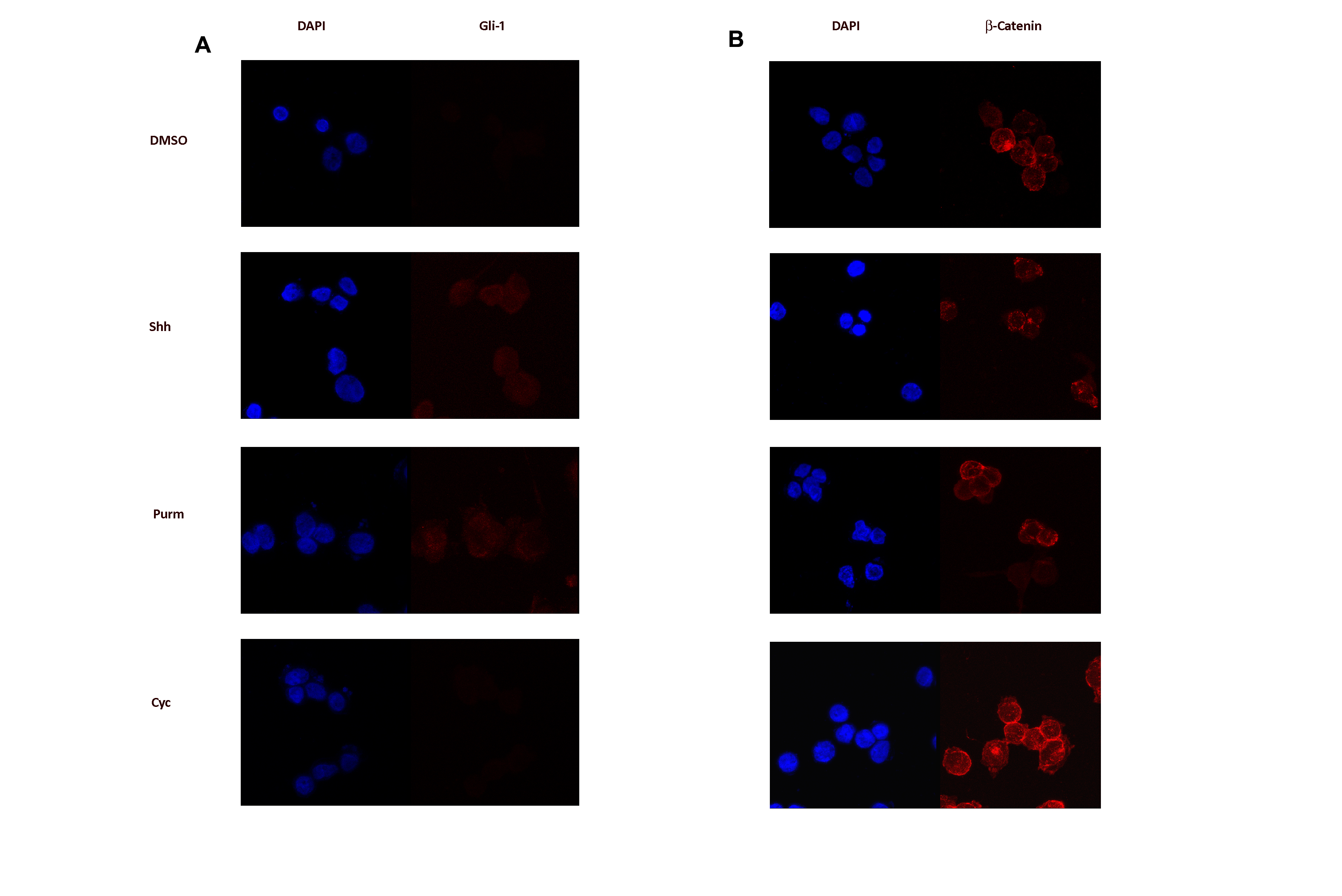

Supplement: Figure S2 — Distribution and levels of Gli-1 and β-catenin in HCT8 cells. Relative nuclear and cytoplasmic distribution and levels of Gli-1 (left panel, A) and β-catenin (right panel, B) in HCT8 cells exposed to different stimuli for 24 hours were analyzed by confocal microscopy. In HCT8 cell, staining densities indicate that Gli-1 protein increases after treatment with Shh (Sonic Hedgehog), or Purm (purmorphamine), compared to cells treated with DMSO (vehicle), or Cyc (cyclopamine). Density of β-catenin decreases upon treatment with Shh compared to either DMSO or Cyc. Nuclei are stained with DAPI (blue). Micrograph panel is representative of 3 experiments for each condition (Original magnification ×1000). (TIF) [file pone.0045332.s002.tif]

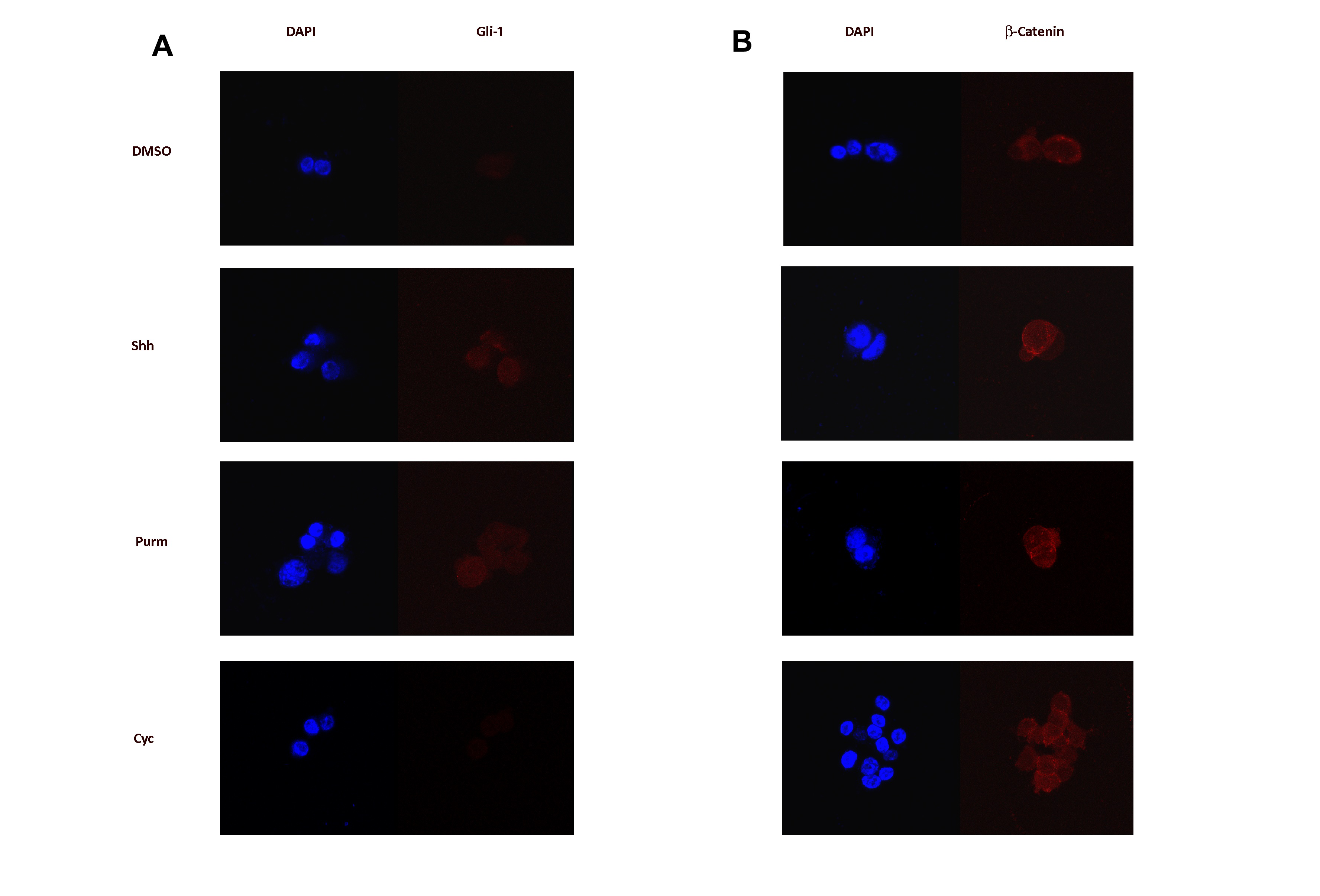

Supplement: Figure S3 — Distribution and levels of Gli-1 and β-catenin in HCT116 cells. Relative nuclear and cytoplasmic distribution and levels of Gli-1 (left panel, A) and β-catenin (right panel, B) in HCT116 cells exposed to different stimuli for 24 hours were analyzed by confocal microscopy. In HCT116, staining densities show that Gli-1 protein increases after treatment with Shh (Sonic Hedgehog), or Purm (purmorphamine), compared to cells treated with DMSO (vehicle), or Cyc (cyclopamine). Density of β-catenin decreases upon treatment with Shh compared to either DMSO or Cyc. Nuclei are stained with DAPI (blue). Micrograph panel is representative of 3 experiments for each condition (Original magnification ×1000). (TIF) [file pone.0045332.s003.tif]

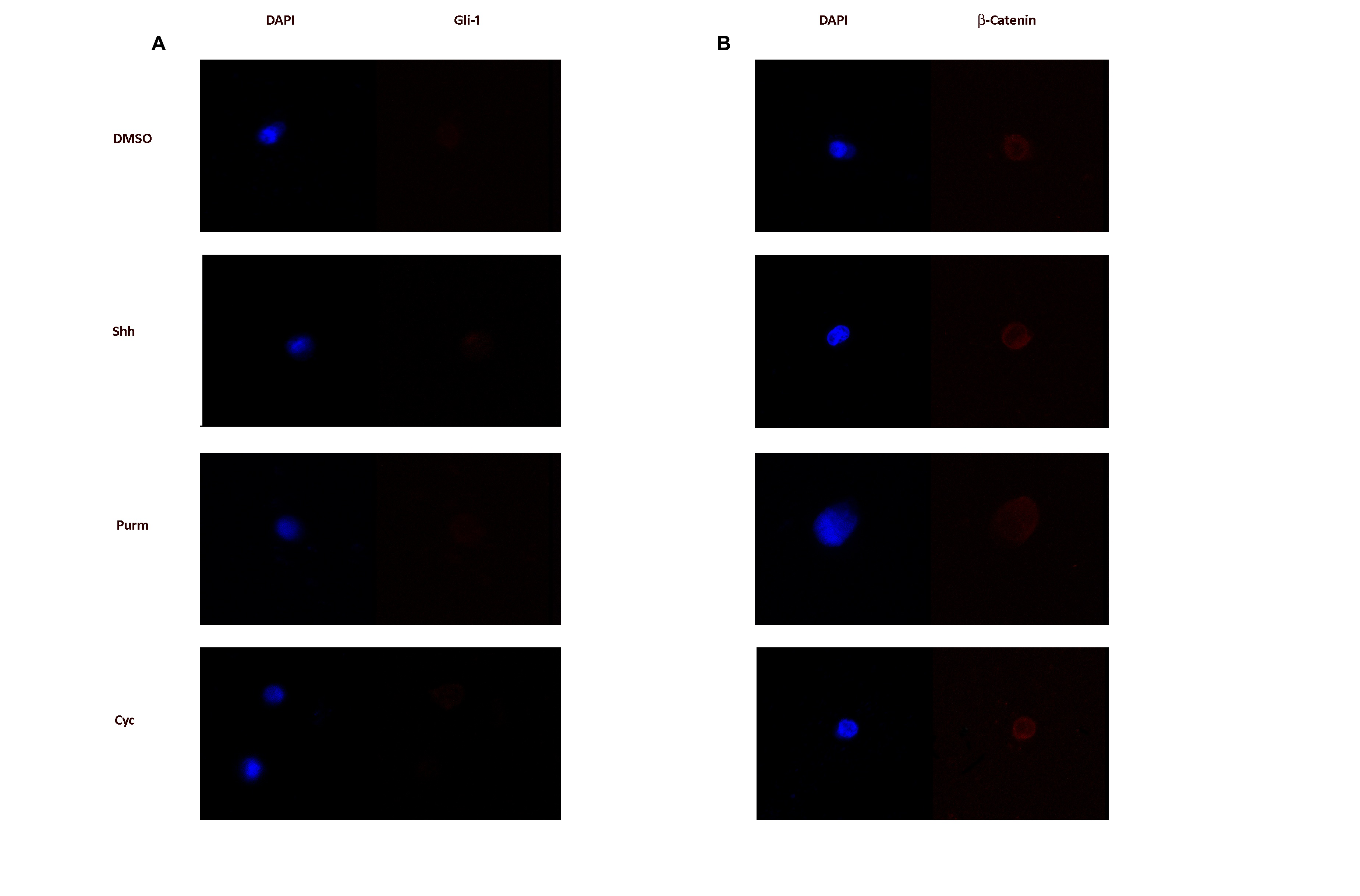

Supplement: Figure S4 — Distribution and levels of Gli-1 and β-catenin in Caco-2 cells. Relative nuclear and cytoplasmic distribution and levels of Gli-1 (left panel, A) and β-catenin (right panel, B) in Caco-2 cells exposed to different stimuli for 24 hours were analyzed by confocal microscopy. In Caco-2 cells, staining of Gli-1 protein is almost undetectable and do not change after treatment with Shh (Sonic Hedgehog), Purm (purmorphamine), Cyc (cyclopamine), compared to cells treated with DMSO (vehicle). Densities of β-catenin are low and do not change upon treatment with Shh or Cyc, compared to DMSO. Nuclei are stained with DAPI (blue). Micrograph panel is representative of 3 experiments for each condition (Original magnification ×1000). (TIF) [file pone.0045332.s004.tif]

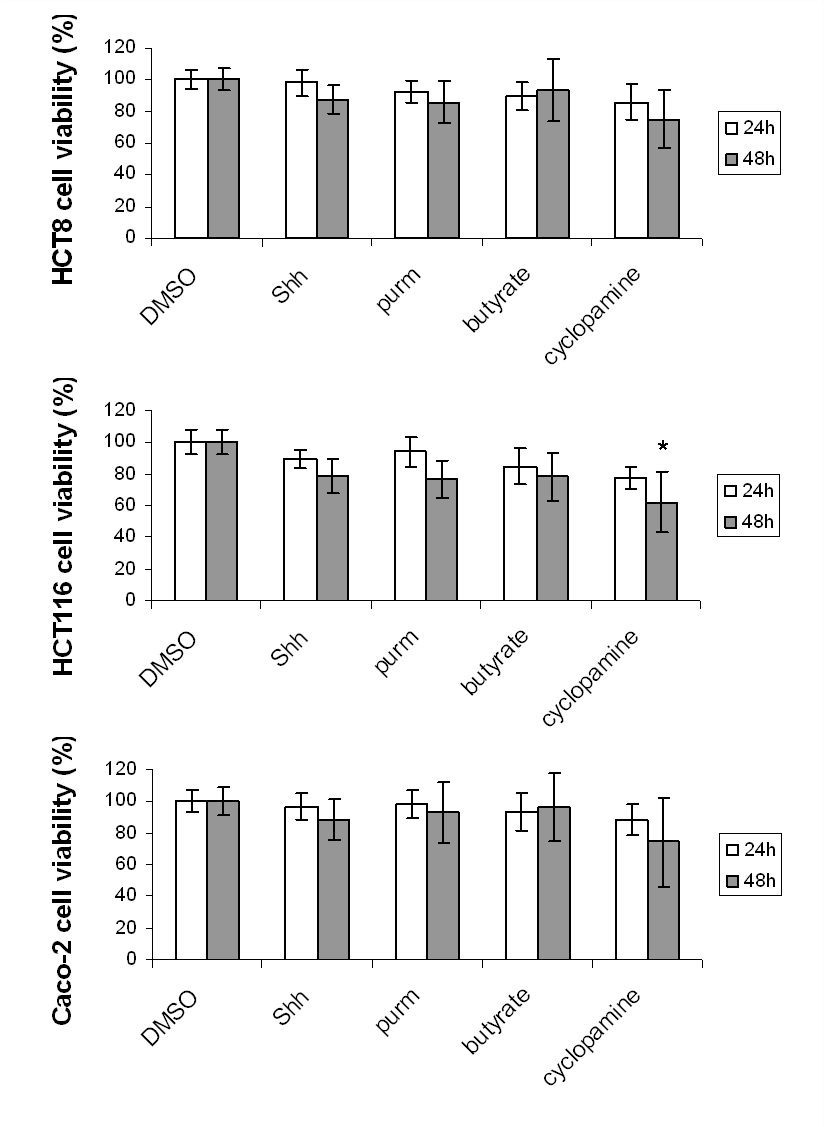

Supplement: Figure S7 — Survival of HCT8, HCT116, and Caco-2 cells upon exposure to different stimuli. Cell viability was analyzed at different time points with different treatments, using the MTT assay. When HCT116 cells were exposed to cyclopamine, cell viability decreased significantly comparing 48 with 24 hours (*P<0.04). Data are expressed as the mean ± SEM of 3 independent experiments each. (TIF) [file pone.0045332.s007.tif]

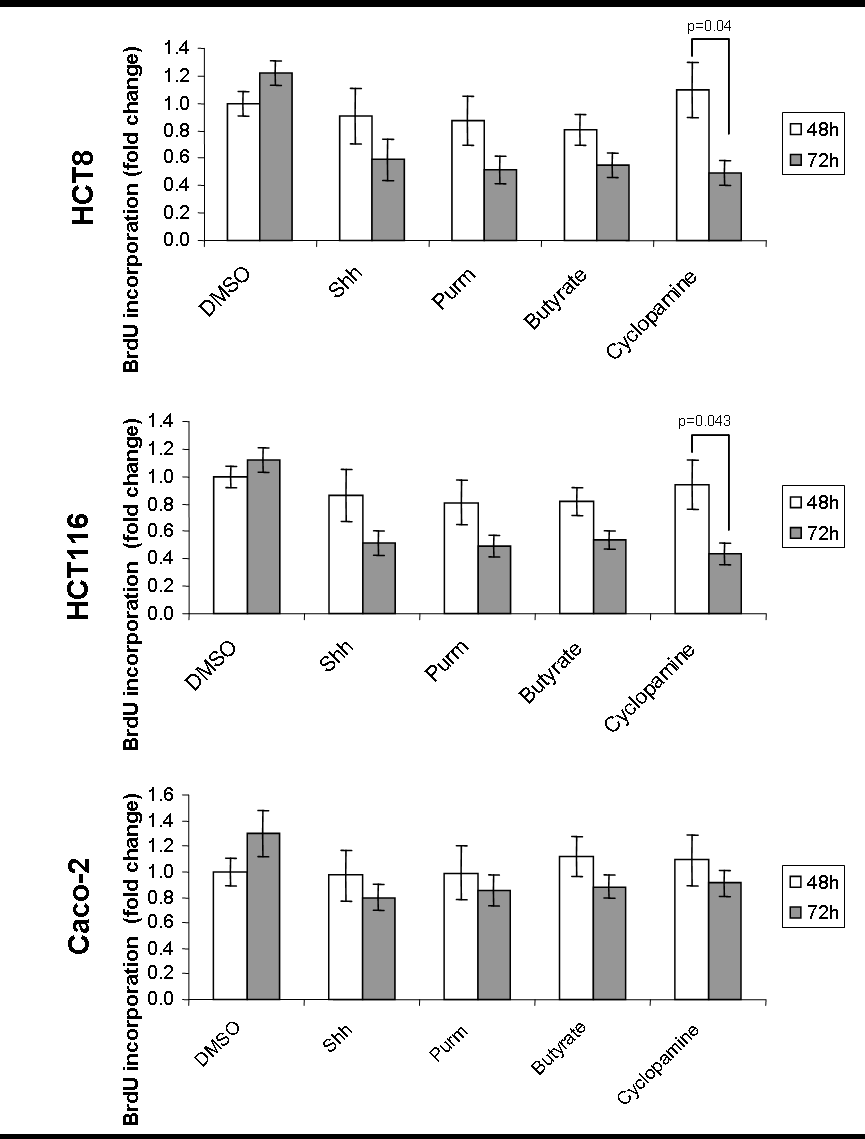

Supplement: Figure S8 — Proliferative activity of HCT8, HCT116, and Caco-2 cells upon exposure to different stimuli. Changes in the proliferative activity of cells were analyzed with different treatments, using the cellular incorporation of BrdU, measured at 48 and 72 hours. A significant decrease in BrdU incorporation was observed from 48 to 72 hours within the group of cyclopamine exposed HCT8 (P<0.04) and HCT116 (P<0.043) cells, respectively. Data are expressed as the mean ± SEM of 3 independent experiments each. (TIF) [file pone.0045332.s008.tif]

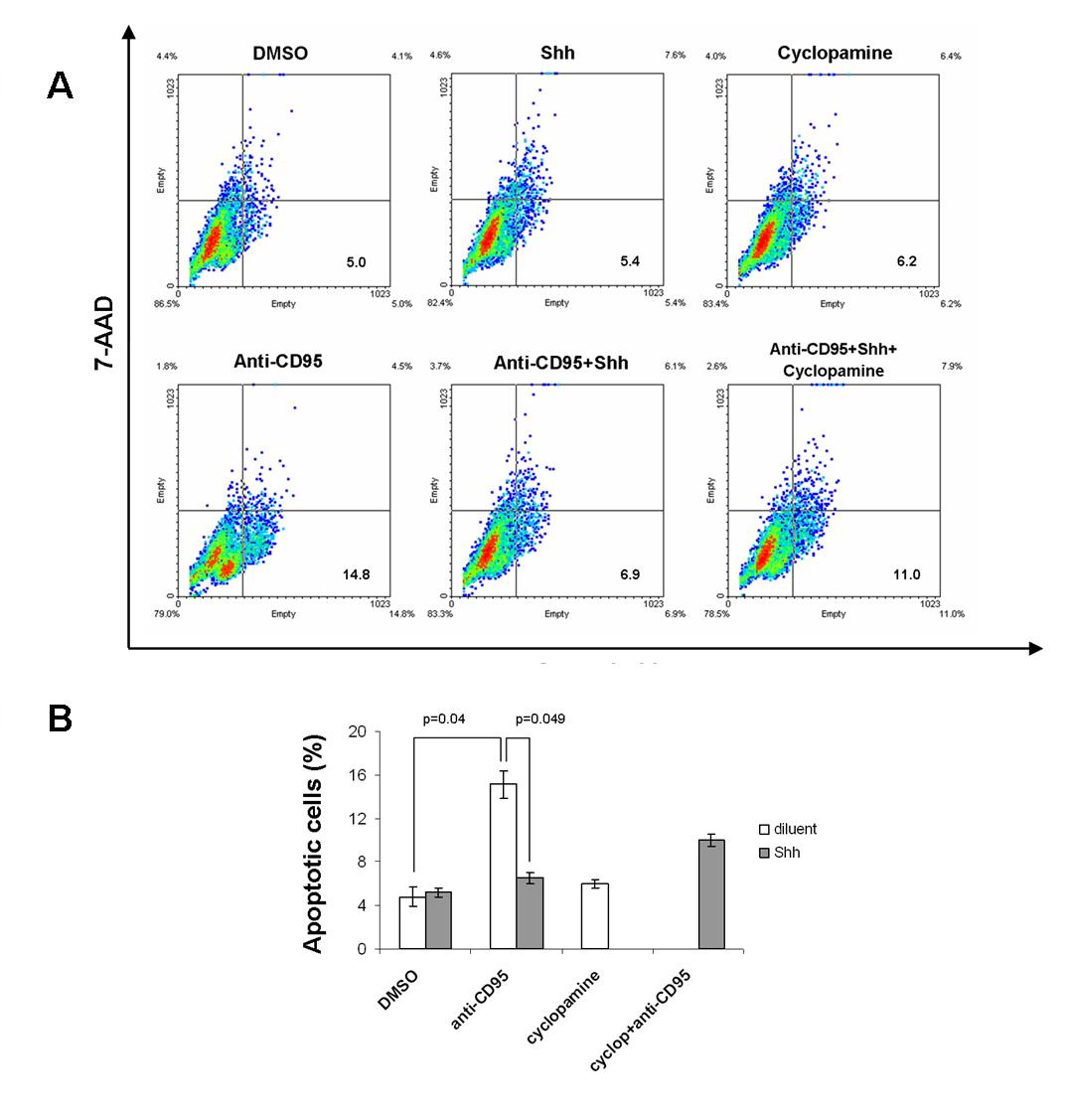

Supplement: Figure S9 — Relationship between apoptosis and the activity of Hedgehog pathway in HCT8 cells. Flow cytometric demonstration of cell apoptosis after 24 hours of exposure to different stimuli, as assessed by annexin-V/7-AAD. Left lower quadrant indicates double negative cells while right lower quadrant indicates annexin-V-positive cells (A). For HCT8 cells, treatment with rShh significantly inhibited the anti-CD95 induced apoptosis (P<0.049), which is partially restored by the addition of cyclopamine (B). Data are expressed as the mean ± SEM of 3 independent experiments. (TIF) [file pone.0045332.s009.tif]

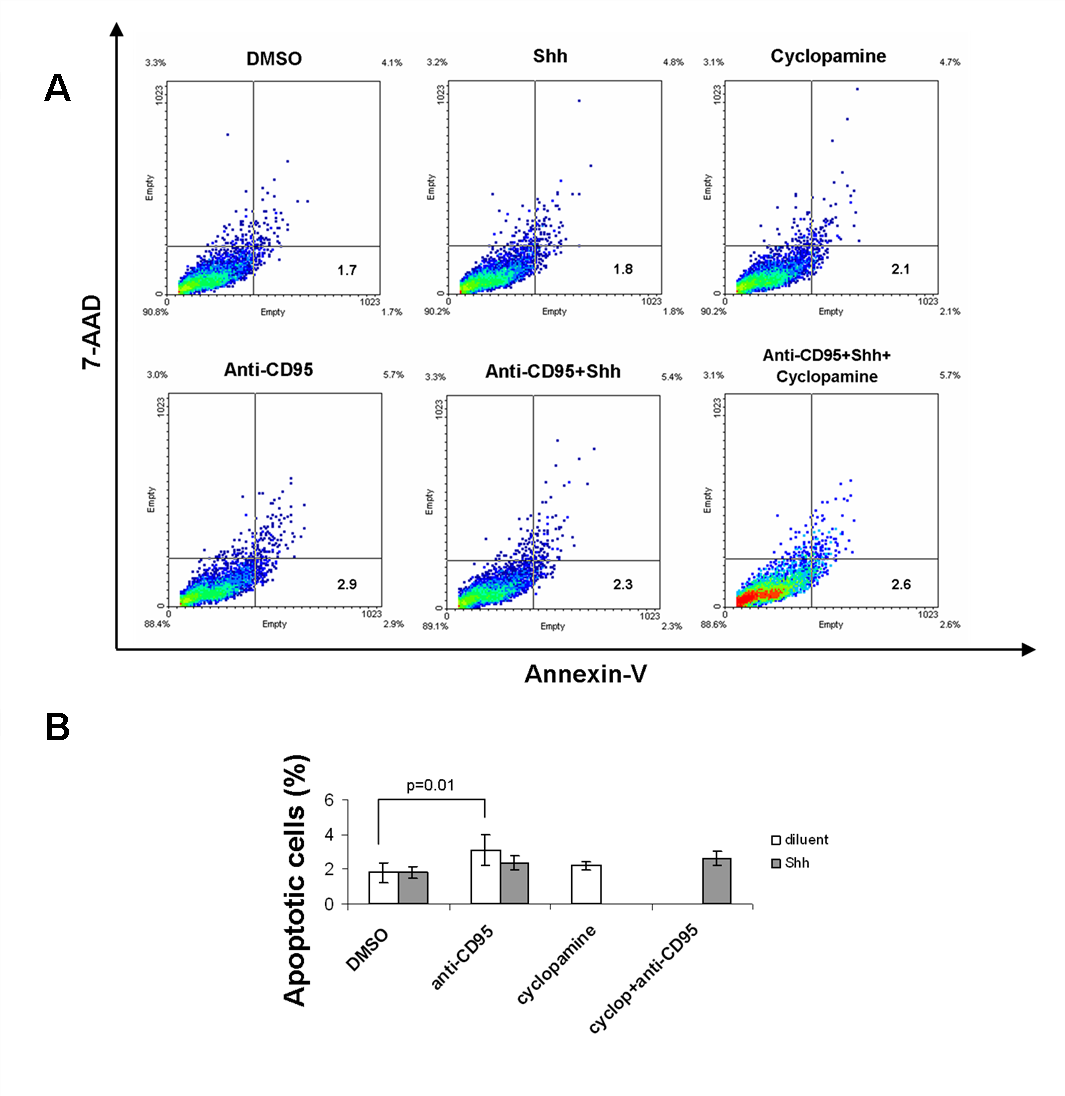

Supplement: Figure S10 — Relationship between apoptosis and the activity of Hedgehog pathway in Caco-2 cells. Flow cytometric demonstration of cell apoptosis after 24 hours of exposure to different stimuli, as assessed by annexin-V/7-AAD. Left lower quadrant indicates double negative cells while right lower quadrant indicates annexin-V-positive cells (A). For Caco-2 cells, anti-CD95 mediated cell death was not significantly modified by rShh or cyclopamine (B). Data are expressed as the mean ± SEM of 3 independent experiments. (TIF) [file pone.0045332.s010.tif]
