# Supplementary material for: Hedgehog Pathway Signaling Regulates Human Colon Carcinoma HT-29 Epithelial Cell Line Apoptosis and Cytokine Secretion
Source: PLoS One. 2012 Sep 19;7(9):e45332. doi: 10.1371/journal.pone.0045332 (PMC3446889; doi:10.1371/journal.pone.0045332)
Supplement: Supporting Information S1 — Supporting Information. (DOC) [file pone.0045332.s011.doc]

**Supporting Information S1**

**Materials and Methods**

**Cell cultures**

Human colon adenocarcinoma cell lines HCT8, HCT116 and Caco-2 cells were obtained from the American Type Culture Collection (ATCC, Rockville, MD, USA), and maintained according to the ATCC’s instructions. Cells were grown as monolayers in 25 cm^2^ culture flasks with DMEM supplemented with heat inactivated 10% FBS and antibiotics as described in Material and Methods session. After growth to confluence, cells were trypsinized and resuspended in DMEM, and seeded in different plates according to the needs of each experiment. All experiments shown with HT-29 cells were performed in parallel using the above mentioned cell lines, maintaining basically the same conditions.

**Indirect immunofluorescence staining and confocal laser microscopy**

Cells were seeded onto eight-chamber slides, fixed, permeabilized, and incubated with blocking solution and appropriate primary and secondary antibodies, as described for HT-29 cells. Expression and localization of the proteins were observed with a confocal laser scanning microscope.

**Quantitative Real-Time PCR (qRT-PCR)**

To quantify the changes in mRNA levels, real-time RT-PCR was performed on the ABI Prism 7500 with RT2 Real Time ™ SYBR Green/Rox PCR Master Mix, and using the same parameters as previously described for setting up the experiments and analyzing data. For these complementary experiments, cell lines were treated during 24h in triplicate with rShh, cyclopamine, or vehicle (DMSO).

**Chemokine Measurements**

Culture supernatants were used for measuring the extra cellular concentration of IL-8 and MCP-1 by ELISA, under exactly the same conditions applied to HT-29 cells.

**Assessment of cell proliferation and viability**

For the assessment of cell proliferation and cell viability, we used the BrdU Cell Proliferation Assay Kit and the MTT assay, respectively, using exactly the same treatment conditions as it was shown with HT-29 cells.

**Assessment of apoptosis**

Cell lines were treated with anti-CD95/APO-1 monoclonal antibody to induce apoptosis, or vehicle control (DMSO), in triplicate, for 24 hr, as described previously for experiments with HT-29 cells. Early and late-stage apoptosis were assessed by flow cytometry, using the Annexin-V/7-AAD Kit.

**Results**

**Expression and modulation of Hh pathway components in HCT8, HCT116 and Caco-2 cells**

We analyzed the effect of stimulating or inhibiting the Hh pathway, and the exposure to different stimuli, in the gene expression of colon cancer cell lines.

Levels of *IHH* and *SHH* mRNA basically have not changed with either the exposure to rShh-peptide or to cyclopamine. However, a tendency for upregulation was detected in both HCT8 and HCT116, in particular for *IHH* in HCT116 (*P* = 0.07). In regard to Gli transcription factors, treatment with rShh-peptide significantly increased mRNA levels of *GLI-2* in HCT8 cells (*P* = 0.03), and of *GLI-1* in HCT116 cells (*P* = 0.04). Levels of *GLI-3* did not change significantly, in none of the cells. The expression of *PTCH1*, *SMO*, and *HHIP* did not change significantly in HCT8, HCT116 and Caco-2 cells upon exposure to rShh-peptide or cyclopamine. The expression of *WNT1* has not changed significantly, however a tendency for increase was detected among HCT116 and especially in HCT8 cells (*P* = 0.06) following exposure to cyclopamine. A tendency for opposing effects of rShh-peptide and cyclopamine were apparently detected again among HCT8 and HCT116 in respect of bone morphogenetic proteins (BMPs). Significant increase with Shh (*P* = 0.028), and decrease with cyclopamine (*P* = 0.014) was shown only for *BMP4* within HCT116 cells. In Caco-2 cells, none of the genes analyzed were affected by the exposure to rShh or cyclopamine (Figure S1).

**Subcellular levels and distribution of Gli-1 and β-catenin in HCT8, HCT116, and Caco-2 cells**

Gli-1 protein was present in HCT8 (Figure S2) and HCT116 (Figure S3) cells, while only very low levels were detected in Caco-2 cells (Figure S4). Similar to the effects observed with HT-29 cells, when HCT8 and HCT116 cells were exposed to rShh-peptide or purmorphamine, levels of Gli-1 increased, whereas an opposing effect was shown after cyclopamine. A constitutive expression of β-catenin was detected in HCT8 and HCT116 cells, while low levels were shown in Caco-2 cells (Figures S2, S3 and S4). Exposure of HCT8 and HCT116 cells to Hh agonists reduced the expression of β-catenin (restricted to the membrane).

**Effect of Hh pathway on IL-8 and MCP-1 secreted by HCT8, HCT116, and Caco-2 cells**

In HCT116 cells, levels of IL-8 were significantly lower following treatment with either rShh-peptide, purmorphamine, or butyrate compared to those treated with cyclopamine (**P* < 0.04) (Figure S5). MCP-1 production also decreased significantly in HCT116 cells treated with rShh-peptide, purmorphamine, or butyrate compared to the ones exposed cyclopamine (**P* = 0.037), to LPS (***P* = 0.043), or IFN-γ (****P* < 0.049), respectively (Figure S6).

In HCT8 cells, levels of MCP-1 were significantly lower upon exposure to either rShh-peptide, purmorphamine, or butyrate compared to those treated with cyclopamine (**P* < 0.04). Data are expressed as the mean ± SEM of 3 independent experiments (Figure S6).

**Effect of Hh pathway on survival and proliferative activity of HCT8, HCT116, and Caco-2 cells**

No significant difference was detected among the cell lines during the first 24 hours. Nevertheless, cell viability decreased significantly at 48 hours when cells were exposed to cyclopamine (**P* < 0.05) (Figure S7).

A significant decrease in BrdU incorporation was observed from 48 to 72 hours within the group of cyclopamine exposed HCT8 (*P* < 0.04) and HCT116 (*P* < 0.043) cells, respectively (Figure S8). All data in both the MTT and the BrdU assays are expressed as the mean ± SEM of 3 independent experiments each.

**Effect of Hh pathway on CD95-mediated apoptosis in HCT8, HCT116, and Caco-2 cells**

In HCT8 cells, treatment with rShh-peptide significantly inhibited the anti-CD95 induced apoptosis (*P* = 0.049), which was partially restored by the addition of cyclopamine (Figure S9). On the other hand, Caco-2 cells did not show any significant response to rShh-peptide or cyclopamine (Figure S10). Data are expressed as the mean ± SEM of 3 independent experiments.
